# A surveillance sector review applied to infectious diseases at a country level

**DOI:** 10.1186/1471-2458-10-332

**Published:** 2010-06-11

**Authors:** Michael G Baker, Sally Easther, Nick Wilson

**Affiliations:** 1 Department of Public Health, University of Otago, Wellington 23 Mein St, Newtown, Wellington, New Zealand

## Abstract

**Background:**

The new International Health Regulations (IHR) require World Health Organization (WHO) member states to assess their core capacity for surveillance. Such reviews also have the potential to identify important surveillance gaps, improve the organisation of disparate surveillance systems and to focus attention on *upstream *hazards, determinants and interventions.

**Methods:**

We developed a *surveillance sector review *method for evaluating all of the surveillance systems and related activities across a sector, in this case those concerned with infectious diseases in New Zealand. The first stage was a systematic description of these surveillance systems using a newly developed framework and classification system. Key informant interviews were conducted to validate the available information on the systems identified.

**Results:**

We identified 91 surveillance systems and related activities in the 12 coherent categories of infectious diseases examined. The majority (n = 40 or 44%) of these were disease surveillance systems. They covered all categories, particularly for more severe outcomes including those resulting in death or hospitalisations. Except for some notifiable diseases and influenza, surveillance of less severe, but important infectious diseases occurring in the community was largely absent. There were 31 systems (34%) for surveillance of *upstream *infectious disease hazards, including risk and protective factors. This area tended to have many potential gaps and lack integration, partly because such systems were operated by a range of different agencies, often outside the health sector. There were fewer surveillance systems for determinants, including population size and characteristics (n = 9), and interventions (n = 11).

**Conclusions:**

It was possible to create and populate a workable framework for describing all the infectious diseases surveillance systems and related activities in a single developed country and to identify potential surveillance sector gaps. This is the first stage in a review process that will lead to identification of priorities for surveillance sector development.

## Background

The new International Health Regulations (IHR), which came into force in June 2007, require World Health Organization (WHO) member states to assess their core capacity for surveillance and response within two years from this date[1]. Such actions should strengthen the capacity of nations to detect infectious diseases that may represent potential public health emergencies of international concern (PHEIC), notify such events to the WHO, and implement appropriate early interventions [2].

Reviewing surveillance systems for infectious diseases also has the potential to identify worthwhile improvements to the organisation of such systems at a country-level. It may also identify gaps in these systems and whether or not important diseases, hazards, determinants, and interventions are under surveillance at all.

We describe in this article a review of these systems and use the term *surveillance sector review *as this work seeks to identify and examine all of the important surveillance activities across a defined area of disease burden. This approach is therefore distinct from, but complementary to, established methods that concentrate on evaluation of specific surveillance systems [3]. Such methods have often focused on important system attributes such as timeliness [4], sensitivity [5], and ability to detect outbreaks [6], though they have also been applied to evaluation of quite broad disease surveillance systems [7].

A *surveillance sector review *builds upon concepts of more integrated surveillance promoted by the WHO, notably for linking surveillance and action [8]. We broaden this approach to consider hazards, consistent with the Global Burden of Disease focus on risk factors and their surveillance [9] (eg, the STEPS programme for surveillance of risk factors for non-communicable diseases [10]). Our approach is also consistent with frameworks for environmental health surveillance (such as the DPSEEA: Driving-force - Pressure - State - Exposure - Effect - Action framework) that consider the causal web of policies and factors that contribute to health outcomes [11]. Here we attempt to extend the concept of integrated surveillance to an entire disease sector including associated health hazards, determinants and interventions.

In this article we aim to: (i) Present a framework for describing and categorising diverse surveillance systems and illustrate this by applying it to all of the surveillance systems operating for one disease sector in a single developed country (in this case infectious diseases in New Zealand); (ii) Present an approach for systematically reviewing the public health surveillance systems operating across a broad public health sector (a *surveillance sector review*); and (iii) Discuss preliminary findings from this review to illustrate its use for identifying potential surveillance sector gaps that require assessment in later stages of the *surveillance sector review*.

## Methods

### Framework for describing and categorising surveillance systems

Since we could identify no published model or framework in the literature for systematically describing and categorising a full range of surveillance systems, we developed one for this review. We took our basic definition of public health surveillance to be: "the ongoing systematic collection, analysis, interpretation and dissemination of data regarding a health-related event for use in public health action to reduce morbidity and to improve health" [3].

For this review, we interpreted health-related events to include diseases and also *upstream *hazards and determinants as well as interventions that relate to infectious diseases. Diseases were taken to include injury, changes in health status and health outcomes. Hazards were defined as risk and protective factors (including behaviour, population vulnerability, agent characteristics, and exposures) that may affect health through specific, direct causal mechanisms. Determinants were causal factors that may affect health through multiple, often indirect pathways. Interventions were actions taken to control or prevent the occurrence of disease or to minimise its negative health effects.

**Figure 1:**
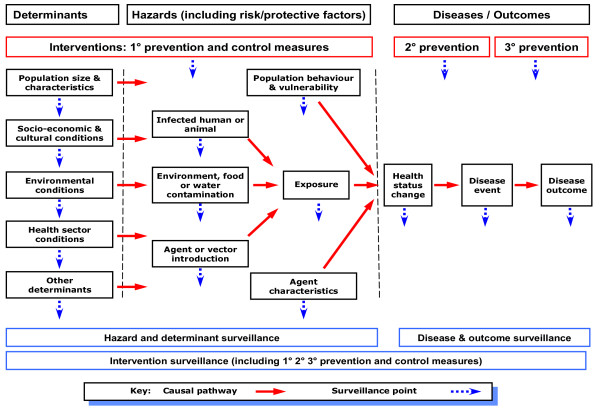
**Framework for classifying events under surveillance (diseases, hazards, determinants and interventions) based on their position along the causal pathway**.

We used the concept of *upstream *to refer to events and factors that were causally related to the disease or other health outcome of interest, and preceded it. For example, these can be proximal hazards, such as exposure to a person with active tuberculosis, or more distal determinants, such as socio-economic position and household crowding [12]. Interventions concerned with prevention may precede disease (primary prevention) or be focused on reducing the effects of disease (secondary and tertiary prevention). The hypothesised relationship between these components of surveillance is shown in Figure 1.

Public health surveillance has multiple aims. To facilitate the functional classification of surveillance systems, we have grouped these aims into two broad purposes: *control-focused *and *strategy-focused *(see Appendix 1). The purpose of *control-focused *surveillance is to identify each occurrence of a particular disease, hazard, or other health-related event that requires a specific response and support delivery of an effective intervention. For example, a single case of polio, a common-source salmonellosis outbreak, a shipment of contaminated produce, or an un-immunised child. The purpose of *strategy-focused *surveillance is to provide information to support prevention strategies to reduce population health risk, such as describing the epidemiology of the annual influenza season and the characteristics of the seasonal influenza viruses. *Control-focused *surveillance usually provides information that can also be used for *strategy-focused *surveillance, so these purposes are often combined within the same surveillance activity. By contrast, *strategy-focused *surveillance cannot generally support *control-focused *surveillance.

As part of this descriptive framework, we categorised surveillance systems into five types:

*• Event surveillance *- defined as "prospective surveillance to identify in a timely manner each occurrence of a particular health-related event, including disease and injury cases, outbreaks, health hazards and interventions".

*• Screening *- defined as "surveillance to identify a particular inapparent disease or pre-disposing risk factor in all members of a specified population, or a particular health hazard in specified settings or environments".

*• Service tracking - *defined as "surveillance to identify delivery and non-delivery of a particular intervention or programme of agreed quality to specified individuals, populations, and settings".

*• Prevalence surveys *- "surveillance based on repeated surveys to measure the prevalence over time of a particular disease or injury, health state, health hazard, health determinant or intervention use in a specified population or setting". It ideally involves a representative sample of the population or setting of interest.

*• Monitoring *- defined as "surveillance based on collection and periodic analysis and interpretation of information to characterise the occurrence and distribution of a particular health-related event, including disease and injury cases, health states, health hazards, interventions, and determinants." It may use data derived from event surveillance, screening, service tracking, or from samples of events (eg, sentinel surveillance, episodic surveillance).

These key terms are consistent with definitions in standard use [12], though they have been adapted for this application to surveillance. In this framework, screening can extend to hazards as in behavioural risk factor screening [13], and environmental screening (eg, screening for resistant organisms in hospitals [14], or microbial contaminants in drinking water [15]).

### Framework for conducting a surveillance sector review

**Figure 2:**
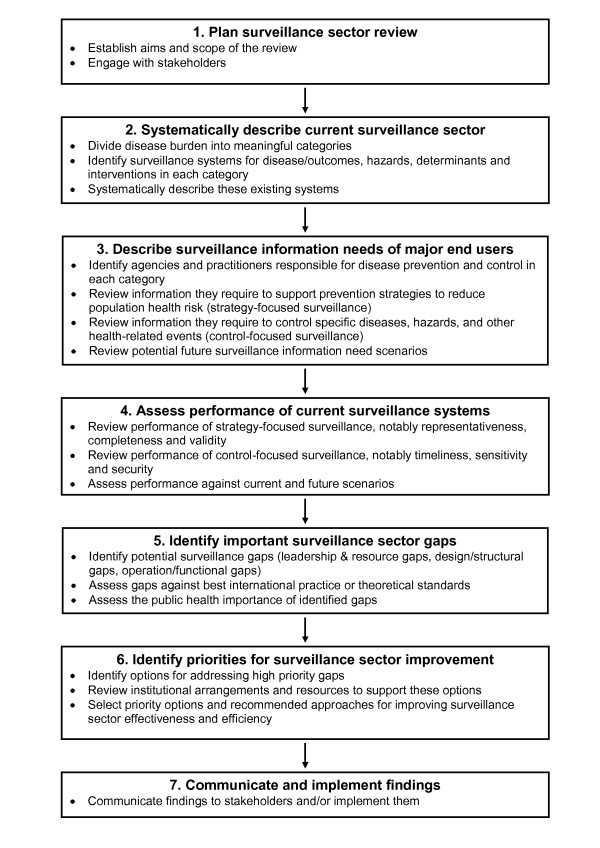
**Framework for carrying out a *surveillance sector review *(note that stage 2 forms the major part of this paper)**.

The framework we have developed for conducting a *surveillance sector review *follows the broad stages shown in Figure 2. We describe each stage in more detail below and refer to how these stages were applied as part of this review work in the New Zealand context.

#### 1) Plan surveillance sector review

It is important to establish the aims and scope of the review. This process will usually be part of negotiating the mandate and resources for the work. Given that public health surveillance is based on a collaborative network of organisations and public health professionals, it is important to engage effectively with the key stakeholders, including end-users and surveillance system operators [3]. We found that this was feasible in the New Zealand setting to the stage reached in this work.

#### 2) Systematically describe the current surveillance sector

To apply this framework to the infectious disease sector, we divided it into meaningful categories. In the New Zealand setting, this step was facilitated by adapting a pre-existing set of infectious diseases categories from a national infectious disease strategy [16]. This process resulted in the following 12 categories based on logical groupings of diseases: vaccine preventable infections; respiratory infections; infections from close physical contact; sexually transmitted infections (STIs); congenital and perinatal infections; blood and tissue borne infections; hospital acquired infections (HAIs) and antibiotic resistance; food borne infections; environmental and water borne infections; zoonotic infections; vector borne infections; and new, exotic and imported infections.

It is then necessary to identify the surveillance systems operating in each category, which we were also able to do for New Zealand (see Additional File 1). In this country many of these systems had a specific name and identity and were managed by a single agency (eg, the notifiable disease surveillance system). Some classification difficulties arose where an agency operated a range of related surveillance activities that shared some common characteristics (eg, the Institute for Environmental Science and Research - ESR - operates several systems for laboratory testing of different groups of microorganisms) or a common information collecting pathway for information that was subsequently analysed and reported on for two different surveillance purposes (eg, the New Zealand Paediatric Surveillance Unit includes acute flaccid paralysis surveillance along with strategy-focused surveillance of a range of paediatric conditions). The resulting listing of surveillance systems could be presented diagrammatically as a surveillance sector map using the framework shown in Figure 1.

The next step is a systematic description of each identified system using the framework described in the previous section (see Additional File 2). In this review we have tabulated for each system the following features: Event under surveillance; Main purpose; Coverage (population or setting); System type; Reporting source; Local/regional collation; and National collation. There is a great deal of additional information which could be used to describe surveillance systems, notably a summary of the system architecture, information collected, resources used, and system performance. We considered this information less critical for this stage of the review for New Zealand, so have not presented it here.

#### 3) Describe surveillance information needs of major end-users

This component describes the surveillance information requirements of end-users. It follows a somewhat analogous approach to the previous section, except that it focuses on information users rather than providers.

The first step is identifying the agencies and practitioners responsible for disease prevention and control in each disease category. These agencies and practitioners include central government end-users (such as the Ministry of Health), regional agencies (such as public health services) and institutional end-users (such as hospital infection control staff), as well as practitioners working at a range of levels inside and outside the health sector. A major focus of this analysis is characterising end-user information needs according to purpose using the same distinction as already noted: *control-focused *surveillance where end-users need to identify and respond to every infectious disease event or scenario (which would include IHR requirements); and *strategy-focused *surveillance where end-users would be asked to define the broad types of strategic information they needed on infectious disease burden to enable them to formulate prevention policies and programmes and measure their effectiveness. An important part of this assessment would be asking end-users to identify their likely future surveillance information needs. This step would be particularly important for identifying uncommon and novel threats that require *control-focused *surveillance.

Information gathering includes interviewing key informants from these agencies and reviewing their infectious disease prevention and control strategies. It may also be useful to review the aims of infectious disease surveillance systems operating in other comparable countries.

#### 4) Assess performance of current surveillance systems

This component is closely aligned to the kind of assessment that would be produced from evaluation of a specific surveillance system [3]. In particular, it seeks to gather empirical data on the performance of each individual surveillance system according to system attributes such as usefulness. The performance of the surveillance systems would be assessed according to the critical attributes required for *control-focused *surveillance (timeliness, sensitivity, stability) and *strategy-focused *surveillance (representativeness, completeness and validity).

Information gathering would ideally be based on reviewing completed evaluations of existing surveillance systems. This evidence base is generally quite limited, so instead would often have to use semi-quantitative assessments based on reviewing the outputs of existing systems and interviews with system operators and users. For *control-focused *surveillance it should usually be possible to estimate timeliness from assessing time-delays in each step of the reporting pathway [4]. Estimating sensitivity may be more difficult and usually requires use of multiple surveillance sources (eg, by a capture-recapture method [5]).

For surveillance of uncommon events and those that have not yet occurred, it would be necessary to use scenario-based exercises to assess system performance. This requirement applies to many of the events that are covered by IHR requirements (where timeliness is probably the single most important system attribute to assess [2]).

#### 5) Identify important surveillance sector gaps

This component identifies high-level deficiencies in existing systems, processes and arrangements. We defined nine types of surveillance sector "gaps" in three broad categories (Table 1). The first category is leadership and resource gaps, which concern the whole sector. They include potential gaps in the leadership and organisational structures to prioritise, develop and coordinate the sector (*leadership and organisational gap*) as well as potential gaps in the workforce and other resources needed to sustain surveillance systems (*workforce gap, resource gap*).

**Table 1:** Classification of potential surveillance sector gaps with illustrative examples

*Gap name*	*Description*	*Example (from the current NZ situation)*
***A. Leadership & resource gaps***		
Surveillance leadership and organisational gap	Lack of sector leadership, coordination, mandate, and supportive organisational structures	No plan for prioritising the development of public health surveillance and limited coordination of existing systems

Surveillance workforce gap	Lack of trained workforce in critical areas, such as epidemiology	Shortage of epidemiologists and data analysts to review and interpret findings from surveillance systems

Surveillance resource gap	Lack of sector resources in critical areas, such as laboratory services and information technology	Limited information technology development resources for public health sector

***B. Design/structural gaps***		

Surveillance system gap	No surveillance system is established for important disease events, *upstream *hazards, determinants or interventions	No surveillance of most common syndromes in primary care (eg, gastroenteritis)

Surveillance scope gap	A surveillance system is established, but its type, range of events covered, and scope of information it is designed to collect does not meet its purpose	Surveillance of hepatitis B and C is largely restricted to acute illness rather than chronic infectious cases

Surveillance coverage gap	A surveillance system is established, but does not cover all of the necessary populations or settings	Absenteeism surveillance and emergency department syndromic surveillance for influenza-like illness is, in each case, confined to only one region in NZ

***C. Operation/functional gaps***		

Surveillance performance gap	An established system doesn't meet necessary requirements for key attributes, such as timeliness, sensitivity and validity	Reporting of hospital discharges and deaths from infectious diseases is complete but not timely, limiting the value of the information for disease control

Surveillance integration gap	Surveillance systems exist but do not link information in a way that supports optimal prevention and control measures	Food and water borne disease surveillance is not routinely linked to drinking water distribution zones so limiting its capacity to detect water treatment failures

Surveillance analysis and communication gap	Surveillance systems operate but do not analyse and disseminate information in a way that supports effective action	Some national data on hospital-acquired infections is collected for health sector monitoring purposes but is not provided to infectious disease practitioners or policy makers

The second category is design and structural gaps, which aim to identify important disease events, hazards, determinants or interventions that are not covered by surveillance systems. This category is concerned with situations where there is no system for surveillance of an important event (*system gap*). Or an established system has not been designed to collect all of the important information to meet its intended purpose (*scope gap*) or is not covering the necessary populations or settings (*coverage gap*).

The third broad category is operation and functional gaps. These gaps include a focus on the performance of a surveillance system (*performance gap*) which is closely aligned to the kind of assessment that would be produced from a specific surveillance system evaluation [3]. We also identified a surveillance *integration gap *where "surveillance systems exist but do not link information in a way that supports optimal surveillance, prevention and control measures". A related type is *analysis and communication gap*, which is concerned with ensuring that information generated by multiple surveillance activities across the sector is being analysed and communicated in an optimal manner to support public health action.

Once these gaps have been identified they would be assessed according to their importance. This assessment would be based on the surveillance information needs identified under stage three above. It is likely that it would be supported by the use of a rating system that categorised gaps according to their importance, distinguishing *control-focused *and *strategy-focused *priority areas. There are approaches for identifying disease surveillance priorities that consider such factors as incidence, impact, preventability and outbreak potential [3]. Burden of disease type analyses would also be useful to highlight the hazard areas contributing most infectious diseases and therefore particularly deserving of surveillance [17].

#### 6) Identify priorities for surveillance sector improvement

This stage seeks to identify the recommended approaches for improving surveillance sector effectiveness. It includes identifying options for addressing high priority gaps and the selection of optimal approaches.

This stage would review the broad institutional arrangements for surveillance and associated activities to identify the extent to which these might need to be modified to achieve the necessary sector improvements. These institutional arrangements include national surveillance functions (eg, surveillance system operation, information collection, integration, analysis, laboratory testing, interpretation, dissemination, aberration detection, investigation, modelling, quality assurance), national response functions (eg, strategy formulation, policy advice, communication, outbreak and epidemic management, emergency response planning and management, linking to other government agencies), national capacity development functions (eg, sector engagement and governance, system planning, evaluation and development, workforce training and development), and regional/local surveillance and response functions (eg, operation of regional/local infectious disease surveillance systems, outbreak detection investigation and management, periodic analysis, reporting and interpretation of infectious disease distribution, integration of data from multiple surveillance sources, development of prevention strategies).

The final part of this stage would be to select priority options and recommended approaches for improving surveillance sector effectiveness. This process would need to include a high level of engagement with the surveillance sector across agencies, levels and disciplinary groups.

#### 7) Communicate and implement findings

This final stage seeks to communicate the findings of the review to stakeholders. Depending on the mandate, the review might extend to implementing the findings. For the New Zealand situation we were able to report findings to the Ministry of Health and also aim to reach the broader health sector in this country through describing some of the findings in this article.

### Application of surveillance sector review to infectious disease sector in New Zealand

#### County selection

New Zealand was selected for this review because of the authors' in-depth familiarity with its systems and because this country is small and organisationally simple for a developed country (ie, it has no state or provincial governments and no agencies at the national level with significantly competing roles). Furthermore, infectious diseases remain an important concern for this country's health sector given the role they play in morbidity and mortality and the absence of an overall decline in incidence or health impact [18,19].

#### Search strategy for the New Zealand systems

We carried out an extensive review that aimed to identify all the current infectious disease surveillance systems in New Zealand. A literature review was performed by searching articles in Ovid Medline using MESH headings and Google Scholar, using search terms such as "surveillance", "infectious disease" and "New Zealand". Articles from 1 January 1990 to December 2009 were included in the search. The search also covered key websites of national surveillance providers such as the website of ESR and the websites of surveillance end-users such as those of the Ministry of Health, Department of Labour, Ministry for the Environment, Pharmac, Medsafe, Statistics New Zealand, New Zealand Food Safety Authority and Ministry of Agriculture and Forestry (which includes Biosecurity New Zealand). Information was mainly extracted from reports found on these websites, however some information was also found directly from the websites themselves. Information was also found by referring to key infectious disease guidelines such as the Ministry of Health's "Communicable Disease Control Manual" [20], and the "Manual for Public Health Surveillance in New Zealand" [21]. As the information was found, it was summarised in tabular form to identify and describe the systems.

#### Key informant interviews in New Zealand

Once an extensive review had been performed, key informants with expertise in infectious diseases in New Zealand were interviewed either by telephone (n = 6) or face-to-face (n = 12) to clarify and confirm the information gathered. Informants were either academics, working for agencies that run infectious disease surveillance, or specialised clinicians involved in the infectious disease part of the health sector. At least one informant specialising in each of the 12 infectious diseases categories was interviewed and each was asked to critically review the information related to their area of expertise. They were also asked whether there were any systems that had not been identified for disease, hazard, determinant or intervention surveillance. The final draft of the document was also reviewed by 13 of the same informants. Results were further checked by the authors through direct inquiries with public health workers and surveillance system operators and review of published material.

The final revision of the manuscript for this article benefited from discussions that took place as part of a subsequent Ministry of Health review of New Zealand's infectious disease surveillance capacity.

## Results

### Infectious disease surveillance systems identified

For the 12 coherent categories of infectious diseases examined, we identified 91 systems and related activities for surveillance of diseases, hazards, determinants and interventions. These systems are shown in Additional File 1 and listed in more detail in Additional File 2. As noted in the Methods Section, some of these activities are not formally described as public health surveillance systems but do provide essential ongoing information (eg, the five-yearly population census). There is also potential to combine or sub-divide some of these systems (eg, the notifiable disease surveillance system). Consequently, the final total of systems is somewhat arbitrary.

We identified 40 systems (44% of the total) for surveillance of infectious diseases and outcomes. All 12 infectious disease categories were covered, to varying degrees. Several systems monitored disease outcomes in multiple categories, such as the Mortality Data Collection (deaths)[22], National Minimum Data Set (NMDS) (hospitalisations)[23], the Notifiable Disease Surveillance System[24], New Zealand Paediatric Surveillance Unit (uncommon infections in children)[25,26], Notifiable Occupational Disease Surveillance System (infections acquired at work) [27], and outbreak surveillance[28]. In addition, most disease categories also had specialised surveillance systems for specific diseases, notably influenza [29-31], HIV/AIDS [32,33], sexually transmitted infections (STIs) [34], transfusion related infections [35], Creutzfeldt-Jakob disease (CJD) [24], and HAIs [36,37].

The surveillance of *upstream *hazards for infectious diseases used a further 31 identified systems (34% of the total). All categories of infectious disease had hazards that were covered, except for infections from close physical contact. These systems could be considered in several ways, including the types of hazards under surveillance. Several systems (n = 4) focused on surveillance of microbial agents and their characteristics, notably antimicrobial resistance surveillance of organisms from humans [38] and food animals [39] and surveillance of agents in relation to vaccine composition [40,41]. Seven systems focused on behavioural factors (knowledge, attitudes, practices) influencing infectious disease risk. These factors included sexual behaviour [42,43], tobacco use [44], food handling [45], hand washing by healthcare staff in hospitals [46], hand sanitiser use by hospital visitors [47,48], and registration of skin piercing practitioners [49]. Two systems focused on identifying infected people who might pose an infectious disease risk to others, including hospitalised patients [36] and pregnant women [50-52]. Only one system specifically measured exposure incidents, and that was for needle-stick injuries (although data were not collated nationally).

Five systems were concerned with surveillance of contamination levels of food [53-56] and drinking water[57]. Four systems were concerned with surveillance of specific environmental events and contaminant levels in a range of settings, including recreational water [58], and less developed systems for cooling towers, swimming pools, and hospital operative equipment.

There were a total of five systems concerned with surveillance of zoonotic infections that might also pose a risk to human health [59-64]. We identified a further three systems concerned with borders and introduction of new pathogens and their vectors (focusing on detection of mosquito vectors [65,66], arboviruses [67], and other pests and new organisms [68]).

We have listed six activities that generate data on the size of the potentially exposed population. On their own, these activities are not surveillance systems but are included in this review because of the critical information they provide for surveillance purposes. For example, the five-yearly census provided population data for most infectious disease categories [69]. These data were supplemented by more specialised sources where specific sub-populations were relevant, notably for births [70], overseas travellers [71], and the hospitalised population[23]. Apart from providing basic demographic data, these data sources generally did not provide much information about the characteristics of those populations that could be used to assess risk and protective factors. A recent addition is New Zealand Health Tracker, which combines data from multiple national data collections, along with primary health organisation (PHO) enrolments, to provide denominator populations that can be analysed according to chronic disease status.

In addition to population size and characteristics, we identified three other determinant areas where information was being gathered. These areas were socio-economic [72-74], environmental [75], and health sector conditions [76]. All of these areas were monitored and reported on by central government agencies. However, results were not fully integrated into the infectious disease surveillance sector. The one exception was socio-economic position where disease cases could be linked to the deprivation level of their domicile area (via the small area-based deprivation measure: NZDep2006 [77]) allowing the impact of socio-economic inequalities to be measured and monitored over time.

We identified 11 surveillance systems that focused on interventions. Such surveillance was most evident for vaccine preventable infections with four systems for aspects of immunisation coverage [30,78-81], plus adverse events surveillance [82,83], and cold-chain monitoring for vaccines [84,85]. There was a system for surveillance of food borne disease interventions (auditing of food control plans [55]), compliance with drinking water standards [57], and the capacity for surveillance of contact tracing and prophylaxis (tuberculosis, meningococcal disease, hepatitis A, and pandemic influenza).

### Systematic description of current surveillance systems

The key features of all 91 identified infectious diseases surveillance systems and related activities are systematically described in Additional File 2. All the systems had a specific event(s) under surveillance. The disease surveillance systems (n = 40) mostly monitored disease outcome by measuring disease incidence. The hazard (n = 31), determinant and population (n = 9) and intervention (n = 11) surveillance systems varied in the type of event they were monitoring. For example, *upstream *surveillance for vaccine preventable infections focused on vaccine coverage (which we have classified as an intervention, but which could also be a hazard if the focus is on the risk posed by under-immunised populations) [78,80].

We classified 44 systems (48%) as exclusively *strategy-focused*, 11 systems (12%) as *control-focused*, and 36 systems (40%) as meeting both *control *and *strategy *purposes. There was a tendency towards more *control-focused *surveillance of hazards and interventions than was seen for diseases and determinants.

Each surveillance system had a set population or setting it aimed to cover. For many of the disease surveillance systems this was the total population, however some systems were aimed at specific subgroups (eg, the Notifiable Occupational Disease System covers only those who are employed [27]). Most of the hazard surveillance systems covered only a sample of the population or settings. The surveillance of determinants aimed to cover a representative sample of the whole population or specific environments, using the census and large-scale national surveys and data collections. Coverage for the surveillance of interventions depended on the intervention. Since most interventions aimed to reach an entire population, the associated surveillance activity was similarly based (and depended on a representative sample of the target population, or all individuals in that population if it was tracking delivery of the intervention).

The identified surveillance systems varied in their type (as defined in the Methods Section). Disease surveillance was generally based on *event surveillance *(n = 15) and *monitoring *(n = 15) of incident cases. There was less use of active methods such as *screening *(n = 5) and *prevalence surveys *(n = 4). Multiple system types were used in some environments (eg, HAI surveillance in hospitals). Hazard surveillance was more reliant on active information collection processes, notably *screening *(n = 10), and periodic *prevalence surveys *(n = 6), though still used *event surveillance *(n = 4) and *monitoring *(n = 3). Determinant surveillance, including measuring the size and characteristics of the population, used *monitoring *(n = 4), periodic *prevalence surveys *(n = 3), and combinations of the two (n = 2). Intervention surveillance tended to use *service tracking *(n = 7), with far less use of *monitoring *(n = 2), and *prevalence surveys *(n = 1).

Surveillance systems require data collection, collation and analysis. Key features of the system architecture are summarised in Additional File 2, which lists the reporting source, local/regional collation, and national collation. Occasionally the same group undertook all three tasks within a surveillance system eg, some surveillance by the New Zealand Food Safety Authority [53-56], and Biosecurity New Zealand [59-63,86,87]. But usually data were reported by different individuals from those who performed the collation and analysis.

Most disease surveillance systems, not surprisingly, were dependent on clinicians recognising and reporting cases. There is a legal requirement to report certain diseases (eg, the notifiable disease system[24]), while others are reported voluntarily (eg, to the New Zealand Paediatric Surveillance Unit [25,26]). Hazard surveillance involved a much wider range of reporting sources, which tended to be associated with a particular activity or product, often monitored at the industry level and only scrutinised at the national level periodically or if an outbreak occurs. Determinant surveillance was run at a national level by relevant government agencies. Intervention surveillance was run by varying groups depending on the specific intervention under surveillance.

About one third of data collation and analysis included a local or regional level prior to transmission to a national-level agency. This is a key role for public health services where such data are used to guide *control-focused *actions such as contact management and outbreak investigation and control. This function also happens at an institutional level, such as for District Health Board (DHB) infection control surveillance. At the national level, collation functions were often performed by groups within the relevant policy agency (notably Ministry of Health, Ministry of Agriculture and Forestry, Department of Labour, Accident Compensation Corporation, Ministry for the Environment). There were also several specialised surveillance agencies and units that performed these national collation, analysis and reporting functions on contract to the Ministry of Health (notably ESR, New Zealand Paediatric Surveillance Unit, AIDS Epidemiology Group, Centre for Adverse Reactions Monitoring). In the case of Statistics New Zealand, the collection, analysis and dissemination of robust data are its core business activities.

### Potential gaps in infectious disease surveillance

The first major stage of a *surveillance sector review*, presented here, has provided a systematic description of the infectious disease surveillance sector operating in New Zealand. On its own, this description cannot identify important surveillance sector gaps. That subsequent stage (see Figure 2), requires careful description of the information needs of the end-users of surveillance outputs, assessment of the performance of current surveillance systems, and then a systematic assessment of the potential gaps identified. However, the systematic description of surveillance systems can identify gaps that are likely to be important. This is particularly the case for design/structural gaps (see Table 1). Some examples of potential gaps are listed below to illustrate the value of this systematic description:

• **Potential surveillance systems gaps: **These gaps occur when there is no surveillance system established for important disease events, *upstream *hazards, determinants or interventions. Assessing such gaps depends on knowledge of which events are important. However, it is reasonable to assume that all disease areas should have sufficient surveillance systems to be able to characterise the disease burden and identify emerging health threats.

All infectious disease categories had systems in place for disease surveillance, at least for severe outcomes like deaths and hospitalisation [22,23]. Two areas appeared to have a comprehensive set of established systems covering diseases, hazards, and interventions. These areas were surveillance of vaccine preventable infections and food borne infections. They included many of the diseases that are prone to outbreaks and are under intensive ongoing surveillance through the notifiable disease surveillance system. Both areas regularly analysed and reported on disease burden and used this information to inform prevention activities [80,88]. At the other extreme, respiratory infections and infections from close contact appeared poorly covered by surveillance systems, with little focus on *upstream *hazards and interventions. Although individual hospitals had systems for surveillance of hospital-acquired infections, there was little aggregation of these data nationally.

Potential surveillance system gaps tended to be for less severe illness at the community and primary care level. There was a particular lack of a well established primary care surveillance system, except for notifiable diseases [24], influenza-like illness [30], and some events under laboratory surveillance [89]. New Zealand has successfully piloted syndromic surveillance in the past for conditions such as gastroenteritis and skin and subcutaneous tissue infection [90]. But such surveillance is now largely absent (except for influenza-like illness [30]).

There were many potential gaps in hazard surveillance. For congenital and perinatal infections, national surveillance of HIV infection in pregnancy has only recently commenced [51,52] and there is little national surveillance of other infectious hazards in this category[91]. Behavioural risk factor surveillance remains limited for STIs [42,43]. The New Zealand Sexual Health Survey, which aimed to collect data on health-related risk and protective behaviours was piloted in 2006, but is currently (as of 2009) either on hold or possibly abandoned. There is little surveillance of hazards for respiratory infections, apart from smoking [92] and no surveillance for factors influencing the risk of infections from close physical contact, such as skin infections. By contrast, some categories of infectious disease included multiple hazard surveillance systems, such as food borne infections [53-56].

There were not many surveillance systems aimed at monitoring disease interventions and therefore many potential gaps. In some instances, such as surveillance of contact prophylaxis and treatment, there is considerable disease control work being carried out by health professionals. However, the surveillance systems have not been fully developed and implemented to capture such data so that it can be used to guide disease control practices and prevention policies.

• **Potential surveillance scope gaps: **These gaps occur where a surveillance system is established, but its type, range of events covered, and scope of information it is designed to collect does not meet its purpose. Some of these potential gaps are apparent from the systematic descriptions presented here.

Some of the surveillance systems identified did not monitor the full range of important events within their scope. An example is latent or asymptomatic disease (eg, HIV, hepatitis B and hepatitis C infection, and some STIs). These forms of infection are poorly covered by most infectious disease surveillance systems, which are orientated towards measuring the incidence of acute clinical infections. Such diseases are often not diagnosed until disease is symptomatic and more serious. A new Public Health Bill has been proposed for New Zealand to classify *Chlamydia *infection, gonorrhoea, HIV infection and syphilis as notifiable diseases [93]. This change should improve STI surveillance and guide prevention efforts to reduce disease.

A further issue around scope arises from the observation that some (n = 11) of the *control-focused *surveillance systems do not supply information to support *strategy-focused *surveillance (eg, serological testing of new prison inmates, immigrant screening for tuberculosis, most antenatal screening for infectious diseases). This problem is particularly common in surveillance of *upstream *hazards in healthcare settings (eg, surveillance of patients colonised with high risk organisms and contaminated operative equipment) and for hazards in the community (eg, surveillance of contaminants in cooling towers and public swimming pools). All of these systems have the potential to supply aggregated data for *strategy-focused *surveillance.

The type of surveillance systems appeared to match their main purpose, at least as described. For example, *control-focused *systems used types of surveillance (ie, event surveillance, screening, and service tracking) that should be able to provide suitable surveillance information to support their purpose. However, it is not possible to say from a description that these systems achieved the necessary performance (eg, in terms of high sensitivity and timeliness).

• **Potential surveillance coverage gaps: **Some surveillance systems identified the appropriate events but did not monitor all of the necessary populations or settings. One example was the clinic-based surveillance of STIs. Data were only reported from participating sexual health clinics, family planning clinics, and student and youth health clinics [34]. Many individuals with STIs see their general practitioner and consequently may not be recorded (except through laboratory-based surveillance which remains incomplete [34]). Consequently, STI surveillance data are far from being representative of the general population.

Another example is the surveillance of refugees and immigrants entering New Zealand. All quota refugees are monitored through the Mangere Refugee Resettlement Centre [94]. However this system misses the refugees entering under family reunification or asylum seekers for whom there is no organised screening programme [95]. Another coverage gap is for HAIs. Data are collected on various HAIs within each hospital and to a certain extent each DHB. However, because the databases and denominator information used within each DHB are different, it is difficult to collate and compare data nationally [36].

## Discussion

### Key findings

As far as we can ascertain, this is the first published attempt to describe the entire infectious disease surveillance sector at a country level. Furthermore, it is also unique in encompassing *upstream *hazards, determinants and interventions along with the more traditional emphasis on disease and health outcomes. This description represents the first major stage in a *surveillance sector review *aimed at identifying high priority improvements to these integrated sector-wide systems.

In this small, developed country, all categories of infectious disease had some form of surveillance, at least for severe outcomes. Most of the large, widely used national infectious disease surveillance systems were aimed at measuring disease outcomes, particularly disease incidence. There were many smaller, narrower systems looking at specific infectious disease hazards. However, these systems generally did not attempt to cover all the hazards in a disease category and thus there are many potential surveillance gaps that require further assessment. The surveillance of interventions was even less well covered with only a small number of systems identified for the whole infectious disease sector. The surveillance of determinants was achieved by large-scale prevalence surveys and ongoing monitoring by national policy agencies.

### Advantages of a surveillance sector review

A *surveillance sector review *approach has some advantages compared with evaluating individual surveillance systems (see Appendix 2). It deliberately 'looks at the forest instead of the trees'. This perspective allows consideration of a wider set of issues than would be covered by evaluation of individual surveillance systems [3]. In particular, it can consider sector development in a more strategic way, and specifically identify gaps in surveillance and areas where investing greater effort could result in public health gain. For that reason, a *surveillance sector review *should be a useful tool to support the assessment requirements of the new IHR which take an all-hazards approach [2].

One generic driver is the need to shift the focus to *upstream *hazards and determinants to provide a surveillance base to support prevention actions. Such a shift requires knowledge of disease causal pathways to identify suitable *upstream *surveillance points.

A related strength of the surveillance sector review approach is that it supports opportunities for greater integration of surveillance activities. Such integration could occur across diseases or between disease and *upstream *hazards, determinants and interventions. One consequence of such integration could be a gain in efficiency if the review process identifies surveillance activities that could be stopped (eg, where there was duplication or where effort could be shifted *upstream *to a more suitable surveillance point).

The categorisation we are using may also provide useful insights when considering opportunities for improved surveillance. Such opportunities may come from deliberately building surveillance activities on existing disease control activities. For example, we identified a number of *control-focused *surveillance activities, such as needle-stick injury surveillance, where these is currently no collation of information for *strategy-focused *purposes. Utilising such information sources is likely to be more efficient than developing separate surveillance systems.

### Disadvantages and limitations of a surveillance sector review

A *surveillance sector review *approach also has disadvantages and limitations (see Appendix 2). By its very nature, this approach provides only a broad overview of the surveillance systems operating across a disease sector. It does not attempt to evaluate the performance of individual systems in depth. That process requires a far more intense focus on individual systems. Methods for such evaluation are well described [3].

This review method also has a number of definitional difficulties, including: establishing the scope of public health surveillance activities; distinguishing these activities from investigation and research; deciding how surveillance activities should be divided or aggregated; and drawing the boundaries of the sector being reviewed (in this case infectious diseases).

The defining features of public health surveillance systems are that the activity is: (i) ongoing, (ii) based on collection and use of information, and (iii) linked to public health action to protect and improve health [3]. Some of the systems included in this review do not currently have all of these features. Because this review process is attempting to identify opportunities for surveillance sector improvement, we took a wide approach to identifying information gathering processes that might contribute to infectious disease control and prevention, even if not currently defined as a surveillance system (eg, inspection and testing of cooling towers for organisms that cause legionellosis). A related issue was whether to include systems that are only operating at a local level, particularly those at the pilot stage. This review has erred on the side of including such systems, particularly where they illustrate an approach that has potential for further development. An example is the pilot sentinel system for influenza-like illness surveillance based in one hospital emergency department [96].

A related boundary is with investigation and research. Given that such activities are not usually ongoing, they are not included as surveillance systems. However, prevalence surveys and other studies can be repeated periodically to provide ongoing information. And many surveillance activities are highly integrated with investigation and research. For that reason, some reviews of surveillance combine these activities with 'studies' as both approaches aim to provide information to guide an effective public health response [97].

Dividing surveillance activities into discrete systems is somewhat arbitrary, so the number of systems listed here is only indicative. For example, organisms collected for public health surveillance purposes, such as *Mycobacterium tuberculosis*, may contribute to both disease surveillance (notably outbreak detection) and hazard surveillance (notably antimicrobial resistance testing), which have been listed as two separate systems. In other areas, particularly determinants, we have grouped a number of surveillance systems into a single named activity. It can also be difficult to categorise some surveillance systems into separate disease, hazard and intervention areas. Effective surveillance of tuberculosis, for example, supports all of these activities. Similarly, surveillance of compliance with food control plans and drinking water standards provides information on the distribution of both hazards and interventions.

The boundary of infectious disease surveillance also becomes less clear as one moves away from systems that are exclusively concerned with surveillance of disease. Most of those involved in surveillance of upstream hazards and determinants would not define their work as infectious disease surveillance. Similarly, some of the health consequences of infectious diseases would not necessarily be included in this sector. For example, surveillance of cervical cancer (including screening) could justifiably be included in this review because this disease is largely an outcome of infection (with human papilloma virus). For these reasons, this review is likely to have under-counted systems contributing to surveillance of infectious diseases.

This review work to date is still just a 'snapshot' of the situation in just one year (2009). For New Zealand, there are important potential developments that are likely to improve surveillance eg, the requirement for direct laboratory notification to local public health services commenced at the end of 2007 and is likely to increase the sensitivity and timeliness of such surveillance [98]. Proposed legislative changes (a new Public Health Bill) would revise the schedule of notifiable diseases by adding STIs (including HIV infection, *Chlamydia *infection, gonorrhoea and syphilis). It would also extend surveillance (notification) requirements to some hazards, notably microbiological contaminants in water cooling towers and drinking water [93].

This analysis is just the first stage in a complete *surveillance sector review*, which is currently (2009) underway in New Zealand. This review will critically assess the potential surveillance gaps identified during this description of the current sector. More importantly, it will assess these gaps against the surveillance information required by end-users for prevention and control of infectious diseases.

The framework used here emphasises the identification of surveillance sector gaps as potential opportunities for system improvements. It is important to also recognise the considerable strengths and successes of the sector being reviewed and the ongoing review and development work already underway.

The approach described in this paper (*surveillance sector review *and the associated descriptive framework) needs to be used more widely to assess its value. It could be applied to the infectious disease surveillance sector in other countries to see if the pattern is comparable. It would also be useful to apply this approach to other disease sectors such as injuries (where surveillance of both outcomes and risk factors is already highly developed [99]), chronic diseases (where risk factor surveillance is particularly well developed for cardiovascular disease [100]), specific hazards (such as tobacco use [101]), and to complex emerging determinants such as climate change.

## Conclusions

We have developed a novel approach for reviewing the full range of surveillance systems required for a single disease sector at a country level (a *surveillance sector review*). As part of this approach we also developed a framework for categorising and describing surveillance systems in a way that highlights their common and contrasting characteristics. This framework appeared useful when applied to describing the infectious disease surveillance systems in a single developed country (New Zealand) and was able to identify potential surveillance gaps for further consideration. This approach and framework could be used to support immediate goals such as assessing surveillance capacity as part of the new IHR requirements. It also provides an approach that could be applied to assessing surveillance of other disease, injury and hazard sectors.

## Competing interests

Two of the authors (MB and NW) were contracted by the Ministry of Health to assist with a Review of New Zealand's Infectious Disease Surveillance Capacity that reported in late 2009. These authors have previously played a role in establishing, running and evaluating some of the surveillance systems described in this review.

## Authors' contributions

MB and NW initiated this study. SE conducted the literature review and key informant interviews and tabulated the key findings. MB and SE drafted most of the paper. All authors read and approved the final manuscript.

## Appendix 1: Purpose and aims of public health surveillance

### A. *Control-focused *surveillance - provides information to support control measures

1. Identify cases, hazards and other health-related events that require a specific response

2. Track delivery, quality, and outcome of specific interventions

### B. *Strategy-focused *surveillance - provides information to support prevention strategies

3. Monitor the occurrence and distribution of disease and injury, including epidemiological, clinical and microbiological features (specific to infectious diseases)

4. Monitor the occurrence and distribution of hazards, risk factors and determinants and support improved prevention measures

5. Monitor the impact of disease, hazards and determinants on the population, health services, and wider society, and help set priorities for prevention and control measures

6. Monitor coverage and effectiveness of interventions and programmes and support their evaluation

7. Support modelling and assessment of future scenarios and interventions

8. Support research, including identifying hypotheses for further investigation

9. Meet legislative and international reporting requirements

10. Monitor the context for surveillance, prevention, and control actions, to guide system development

## Appendix 2. Advantages and disadvantages of a surveillance sector review compared with evaluating individual surveillance systems

### Advantages

• Can identify surveillance sector gaps, notably important public health events that have little or no surveillance

• Shifts focus to *upstream *hazards and determinants that might provide an improved surveillance base to inform and support prevention actions

• Considers a set of issues that may be missed by evaluation of individual systems, notably integration of information from multiple systems

• Identifies sector issues such as leadership, coordination, mandate, organisation, workforce and resources that affect multiple surveillance systems

• Supports assessment of surveillance capacities required for the International Health Regulations 2005 (IHR 2005) which take an *all hazards *approach

• Documents the full range of public health surveillance activities which may facilitate greater use of the information generated

• Provides a more consistent way of describing surveillance methods across diverse types of health events which may assist translation of best practice and improved information quality

### Disadvantages

• Requires considerable time and resources to conduct a comprehensive review of a sector

• Cannot provide detailed assessment of the performance of individual surveillance systems (systematic evaluations of individual systems are still required)

• May raise difficult boundary issues regarding the scope of the sector and the activities that constitute public health surveillance

## Pre-publication history

The pre-publication history for this paper can be accessed here:

http://www.biomedcentral.com/1471-2458/10/332/prepub

## Supplementary Material

Additional file 1 **Infectious disease surveillance systems in New Zealand, classified according to infectious disease category and main event type under surveillance**. A tabulated list of infectious disease surveillance systems is presented, organised according to major infectious disease categories. Click here for file

Additional file 2 **Description of key features of infectious disease surveillance systems identified in New Zealand**. A detailed tabulated list of infectious disease surveillance systems in New Zealand is presented. Information provided includes the system name, the event under surveillance, the main purpose, system coverage and type, reporting source and level of data collation (local, regional and national). Click here for file
