# Supplementary material for: A surveillance sector review applied to infectious diseases at a country level
Source: BMC Public Health. 2010 Jun 11;10:332. doi: 10.1186/1471-2458-10-332 (PMC3224743; doi:10.1186/1471-2458-10-332)
Supplement: Additional file 1 — Infectious disease surveillance systems in New Zealand, classified according to infectious disease category and main event type under surveillance. A tabulated list of infectious disease surveillance systems is presented, organised according to major infectious disease categories. [file 1471-2458-10-332-S1.PDF]

Additional File 1

Infectious disease surveillance systems in New Zealand, classified according to infectious disease category and main event type under surveillance (disease/outcome and hazard/determinant/intervention).

Each named system is described in more detail in Additional File 2.

Note that this list of surveillance systems is likely to be incomplete (see limitations section of paper)

Infectious Disease Category [1]	*Disease/Outcome Surveillance* [2]	*Hazard/Determinant/Intervention Surveillance* [3]
**1. Vaccine-preventable infections**<br>Eg, pertussis, pneumococcal disease, influenza and influenza-like illness (ILI), human papilloma virus (HPV) infection	• Mortality Data Collection (deaths)<br>• National Minimum Data Set (hospitalisations)<br>• Notifiable Disease Surveillance System<br>• Laboratory-based surveillance of measles (for outbreak (OB) detection)<br>• Acute flaccid paralysis (AFP) surveillance (for polio detection)<br>• Sentinel general practice surveillance system and HealthStat (ILI)<br>• Healthline (ILI calls)<br>• Google Flu Trends (ILI)<br>• Syndromic surveillance for ILI<br>• Absenteeism surveillance (workplaces, schools)<br>• Flutracker (ILI)<br>• Intensive care utilisation for influenza<br>• Serosurvey of vaccine-preventable diseases<br>• Outbreak surveillance system	• Exposed population: Census (includes data on those in vaccine-appropriate age groups); NZ Health Tracker<br>• Laboratory-based surveillance of invasive bacterial diseases to support vaccine strategy<br>• Laboratory-based surveillance or influenza to support vaccine strategy<br>• Surveillance of influenza knowledge, attitudes and practices<br>• Determinant surveillance<br>• National Immunisation Register (NIR)<br>• Immunisation coverage surveys<br>• School-based immunisation registers (Immunisation coverage)<br>• Influenza vaccine surveillance<br>• Adverse event surveillance [4]<br>• Cold-chain surveillance<br>• Surveillance of contact prophylaxis and treatment [5]
**2. Respiratory infections**<br>Eg, tuberculosis, rheumatic fever, influenza (see under vaccine-preventable)	• Mortality Data Collection (deaths)<br>• National Minimum Data Set (hospitalisations)<br>• Notifiable Disease Surveillance System<br>• *Mycobacterium tuberculosis* typing (for OB detection)<br>• Outbreak surveillance system	• Exposed population: Census (included data on those living in crowded housing); NZ Health Tracker<br>• Tobacco use surveillance (including active smoking and exposure to second-hand smoke)<br>• Determinant surveillance<br>• Rheumatic fever registers<br>• Surveillance of contact prophylaxis and treatment [5]
**3. Infections from close physical contact**<br>Eg, giardiasis, rotavirus, skin infections	• Mortality Data Collection (deaths)<br>• National Minimum Data Set (hospitalisations)<br>• Notifiable Disease Surveillance System<br>• NZ Paediatric Surveillance Unit (post-streptococcal glomerulonephritis)<br>• Outbreak surveillance system	• Exposed population: Census (included data on those living in crowded housing); NZ Health Tracker<br>• Determinant surveillance<br>• Surveillance of contact prophylaxis and treatment [5]

Infectious Disease Category [1]	*Disease/Outcome Surveillance* [2]	*Hazard/Determinant/Intervention Surveillance* [3]
**4. Sexually transmitted infections (STIs)**<br>Eg, HIV/AIDS, *Chlamydia* infection, gonorrhoea	• Mortality Data Collection (deaths)<br>• National Minimum Data Set (hospitalisations)<br>• Notifiable Disease Surveillance System<br>• HIV/AIDS surveillance<br>• Unlinked anonymous HIV prevalence surveys in sexual health clinic attendees<br>• Clinic-based STI Surveillance<br>• Laboratory-based STI Surveillance<br>• Outbreak surveillance system	• Exposed population: Census (includes data on those in sexually active age-group)<br>• HIV behavioural surveillance among men who have sex with men (MSM)<br>• HIV Futures New Zealand<br>• Determinant surveillance
**5. Congenital and Perinatal infections**<br>Eg, hepatitis B, congenital rubella syndrome, group B streptococcal sepsis of newborn	• Mortality Data Collection (deaths)<br>• National Minimum Data Set (hospitalisations)<br>• Notifiable Disease Surveillance System<br>• NZ Paediatric Surveillance Unit (HIV, rubella, neonatal infections)<br>• Outbreak surveillance system	• Exposed population: Maternity and Newborn Collection (MNIS) (includes new birth registration data on live births, stillbirths and foetuses)<br>• Antenatal infectious disease screening (HIV, rubella, hepatitis B, tuberculosis risk)<br>• Determinant surveillance
**6. Blood and tissue borne infections**<br>Eg, hepatitis C, HIV/AIDS, Creutzfeldt-Jakob disease (CJD)	• Mortality Data Collection (deaths)<br>• National Minimum Data Set (hospitalisations)<br>• Notifiable Disease Surveillance System<br>• HIV/AIDS surveillance<br>• Haemovigilance programme (blood and transfusion-related infections)<br>• National Hepatitis Follow-up Programme<br>• Serosurvey and behavioural risk factor survey of injecting drug users (IDUs)<br>• Serological testing of new prison inmates<br>• CJD surveillance<br>• Notifiable Occupational Disease System (NODS)<br>• Outbreak surveillance system	• Exposed population: Transfusion data (patients requiring blood transfusions, blood products); NMDS (organ transplants, surgery patients); Injecting drug user (IDU) surveys; Healthcare worker registration<br>• Haemovigilance programme (contaminated blood donations)<br>• Registration of skin piercing practitioners<br>• Needle-stick injury surveillance<br>• Antenatal screening (HIV, hepatitis B)<br>• Determinant surveillance
**7. Hospital-acquired infections (HAI) and antibiotic resistance**<br>Eg, surgical-site infections, blood-stream infections, norovirus, methicillin-resistant *Staphylococcus aureus* (MRSA), penicillin-resistant pneumococci	• Mortality Data Collection (deaths)<br>• National Minimum Data Set (hospitalisations)<br>• Notifiable Disease Surveillance System<br>• HAI Surveillance<br>• Laboratory-based surveillance of HAI (for OB detection)<br>• Healthcare facilities antibiotic resistance surveillance<br>• Outbreak surveillance system	• Exposed population: NMDS (patients admitted to hospital, including sub-populations admitted to ICU); Personnel management systems in hospitals (hospital staff); Patient management systems (identification of high risk patients); Healthcare worker registration<br>• Identification of hospital patients colonised with high risk organisms<br>• Surveillance of hospital operative equipment eg, endoscopes<br>• Hand Hygiene New Zealand (HHNZ)<br>• Antimicrobial resistance surveillance<br>• Surveillance of antibiotic use in humans<br>• Determinant surveillance

Infectious Disease Category [1]	*Disease/Outcome Surveillance* [2]	*Hazard/Determinant/Intervention Surveillance* [3]
**8. Food borne infections**<br>Eg, campylobacteriosis, salmonellosis, verotoxin producing *E. coli* (VTEC), norovirus	• Mortality Data Collection (deaths)<br>• National Minimum Data Set (hospitalisations)<br>• Notifiable Disease Surveillance System<br>• Laboratory-based surveillance of food borne diseases (for OB detection)<br>• CJD (atypical) surveillance<br>• Outbreak surveillance system	• Exposed population: Census; NZ Health Tracker<br>• Surveillance of antibiotic resistance in food animals<br>• Surveillance of veterinary medicine (antibiotic) sales<br>• Imported food monitoring programme and Imported food clearance programme<br>• National Microbial Database (Food Production/Processing Surveillance)<br>• Surveillance of marine biotoxins in shellfish<br>• Food handling knowledge, attitudes and practices surveys<br>• Determinant surveillance<br>• Processed and retail food surveillance<br>• Surveillance of food control plans
**9. Environmental and water borne infections**<br>Eg, cryptosporidiosis, legionellosis	• Mortality Data Collection (deaths)<br>• National Minimum Data Set (hospitalisations)<br>• Notifiable Disease Surveillance System<br>• Laboratory-based surveillance of legionellosis (for OB detection)<br>• Notifiable Occupational Disease System (NODS)<br>• Outbreak surveillance system	• Exposed population: Census (can assign population to rural areas, drinking water distribution zones & other spatially referenced environmental exposures); NZ Health Tracker<br>• Natural recreational water surveillance<br>• Cooling tower surveillance<br>• Public swimming pool surveillance<br>• Environmental health indicators surveillance<br>• Determinant surveillance<br>• Drinking water surveillance
**10. Zoonotic infections**<br>Eg, leptospirosis, bovine tuberculosis	• Mortality Data Collection (deaths)<br>• National Minimum Data Set (hospitalisations)<br>• Notifiable Disease Surveillance System<br>• Laboratory-based surveillance of leptospirosis (for OB detection)<br>• CJD (atypical) surveillance<br>• Notifiable Occupational Disease System (NODS)<br>• Occupational disease surveillance (claims database)<br>• Outbreak surveillance system	• Exposed population: Census (occupational groups)<br>• OIE notifiable organisms system (veterinary)<br>• Veterinary laboratory-based surveillance<br>• Avian influenza surveillance<br>• Transmissible spongiform encephalopathy (TSE) disease surveillance<br>• Bovine tuberculosis surveillance<br>• Determinant surveillance
**11. Vector borne infections**<br>Eg, murine typhus, Ross River virus infection	• Mortality Data Collection (deaths)<br>• National Minimum Data Set (hospitalisations)<br>• Notifiable Disease Surveillance System<br>• Laboratory-based surveillance of arboviral diseases (for OB detection)<br>• Outbreak surveillance system	• Exposed population: Census<br>• Arbovirus surveillance<br>• Mosquito surveillance<br>• Determinant surveillance

Infectious Disease Category [1]	*Disease/Outcome Surveillance* [2]	*Hazard/Determinant/Intervention Surveillance* [3]
**12. New, exotic and imported infections**<br>Eg, malaria, dengue fever, anthrax	• Mortality Data Collection (deaths)<br>• National Minimum Data Set (hospitalisations)<br>• Notifiable Disease Surveillance System<br>• Laboratory-based surveillance of arbovirus diseases (for OB detection)<br>• Laboratory-based surveillance of viruses (for OB detection)<br>• Arriving passenger screening<br>• Refugee surveillance<br>• Immigrant screening<br>• Geosentinel network<br>• Outbreak surveillance system	• Exposed population: Census, Arriving passenger survey<br>• Border surveillance<br>• Arbovirus surveillance<br>• Mosquito surveillance

Notes:

[1] Many infectious diseases have multiple modes of transmission and can be placed in more than one category (eg, influenza). Examples are placed in the categories where most transmission is thought to occur or where prevention efforts are focused.

[2] Several surveillance systems collect data on all diseases across all categories (notably mortality, hospitalisation) while other systems collect data on selected diseases across all categories (notifiable diseases).

[3] Several surveillance activities collect data that is relevant to most categories (notably determinant surveillance).

[4] Adverse event surveillance included vaccines, herbal products and dietary supplements for treatments used in any disease category; however the main focus here is on adverse events related to vaccines.

[5] Contact prophylaxis and treatment covers all disease categories but is most relevant to the first three in this table.
