# Supplementary material for: A surveillance sector review applied to infectious diseases at a country level
Source: BMC Public Health. 2010 Jun 11;10:332. doi: 10.1186/1471-2458-10-332 (PMC3224743; doi:10.1186/1471-2458-10-332)
Supplement: Additional file 2 — Description of key features of infectious disease surveillance systems identified in New Zealand. A detailed tabulated list of infectious disease surveillance systems in New Zealand is presented. Information provided includes the system name, the event under surveillance, the main purpose, system coverage and type, reporting source and level of data collation (local, regional and national). [file 1471-2458-10-332-S2.DOC]

Additional File 2

**Description of key features of infectious disease surveillance systems identified in New Zealand**

**Note that this list of surveillance systems is likely to be incomplete (see limitations section of paper)**

| ***System name (or subject area)*** | ***Event under surveillance*** | ***Main purpose*** | ***Coverage (Population or Setting)*** | ***System type*** | ***Reporting source*** | ***Local/ Regional collation*** | ***National collation*** | ***Ref*** |
| --- | --- | --- | --- | --- | --- | --- | --- | --- |
| Surveillance of disease (including health status, disease, injury, outcomes) | | | | | | | | |
| **Multiple types of infection** | | | | | | | | |
| **Mortality Data Collection (deaths)** | Deaths in NZ (including infectious diseases) | Strategy-focused | Total population | Monitoring | Births, Deaths and Marriages (BDM), coroners | Nil | Ministry of Health (MoH) | [1] |
| **National Minimum Dataset (hospital-isations)** | Discharges from hospital (including infectious diseases) | Strategy-focused | All patients admitted to publicly-funded hospitals and larger private hospitals | Monitoring | Hospitals | Nil | MoH | [2] |
| **Notifiable Disease Surveillance System** | Notifiable infectious diseases (approximately 50 diseases and categories) | Control-focused + Strategy-focused | Total population | Event surveillance | Medical practitioners and clinical laboratories | Public health service / Medical Officer of Health | ESR | [3] |
| **Outbreak surveillance system (for OB reporting)** | Outbreaks of infectious diseases (notifiable and some others) | Control-focused + Strategy-focused | Total population (currently excludes outbreaks in hospitals) | Event surveillance | Mostly identified by local public health services and laboratories | Public health service / Medical Officer of Health | ESR | [4] |
| **NZ Paediatric Surveillance Unit (NZPSU)** | Incidence of uncommon infections in children (including acute flaccid paralysis, post-streptococcal glomerulo-nephritis, congenital rubella, perinatal HIV exposure, neonatal infections) | Strategy-focused | Children and infants diagnosed by specialist paediatricians | Monitoring (except AFP – see AFP surveillance) | Participating paediatricians (about 200) | Nil | NZPSU (University of Otago) | [5, 6] |
| **Notifiable Occupational Disease System (NODS)** | Infectious diseases from occupational exposures (eg, leptospirosis) | Control-focused + Strategy-focused | Employed population | Event surveillance | Medical practitioners and individuals | Regional Department of Labour Office | NODS Registrar, Department of Labour | [7] |
| **Occupational disease surveillance (claims database)** | Infectious diseases from occupational exposures (eg, leptospirosis) | Strategy-focused | Employed population | Monitoring | Medical practitioners and individuals | Nil | Accident Compensation Corporation (ACC) | [8] |
| **Vaccine-preventable infections** | | | | | | | | |
| **Laboratory-based surveillance of measles (to support OB detection)** | Viruses obtained from measles cases for confirmation and genotyping of strains | Control-focused + Strategy-focused | Patients with suspected measles | Event surveillance | Laboratories doing serological testing | Nil | MoH | [9] |
| **Acute flaccid paralysis (AFP) surveillance** | AFP cases | Control-focused + Strategy-focused | Infants and children admitted to hospital with AFP | Event surveillance | Paediatricians | Nil | NZPSU (University of Otago) | [6] |
| **Sentinel general practice surveillance system (influenza)** | Influenza-like illness (ILI) and virus collection for strain identification | Strategy-focused | Patients who attend sentinel practices (approximately 90) across NZ, May to September annually | Monitoring | Participating general practices | Public health service / Medical Officer of Health | ESR | [10, 11] |
| **HealthStat (influenza sentinel surveillance)** | Influenza-like illness (ILI) | Strategy-focused | Patients who attend sentinel practices (84 GP practices in original HealthStat panel, additional 300 GP practices recruited in June 2009) | Monitoring | Participating computerised general practices | Nil | MoH | [12, 13] |
| **Healthline (ILI calls from the public)** | Influenza-like illness (ILI) | Strategy-focused | Population who seek health information over the telephone | Monitoring | National Healthline call centre | Nil | MoH | [12, 14] |
| **Google Flu Trends (ILI)** | Influenza-like illness (ILI) | Strategy-focused | Population with Internet access who use this Internet search engine for ILI terms | Monitoring | The US-based company Google.org | Nil | Nil formal mechanism | [15] |
| **Syndromic surveillance for influenza-like illness (ILI)** | Influenza-like illness (ILI) | Strategy-focused | Patients seen at Wellington Regional Hospital Emergency Department | Monitoring | Wellington Regional Hospital Emergency Department | Nil | MoH. Information passed onto Wellington Regional Public Health | [16] |
| **Flutracker (ILI)** | ILI prevalence | Strategy-focused | Nationally representative sample of households (pilot survey) | Prevalence surveys | Subjects interviewed by telephone | Nil | Survey company reporting to MoH | [12] |
| **Absenteeism surveillance systems (workplaces, schools)** | Absenteeism for illness | Strategy-focused | Population attending schools in Otago-Southland (20) and Otago DHB staff (approximately 3000) | Monitoring | Staff running sickness registries of schools, ODHB payroll system | Public health service (Otago-Southland) | Public health service (Otago-Southland) | [12] |
| **Hospital Intensive Care Unit (ICU) influenza utilisation reporting** | Influenza admissions to ICU, occupancy, ventilator capacity | Strategy-focused | All ICUs | Monitoring | Hospital intensive care specialists | DHBs | MoH | [12] |
| **Serosurvey of vaccine-preventable infections** | Sero-prevalence of antibodies to vaccine preventable infections, including influenza | Strategy-focused | Sample of adults and children | Prevalence surveys | Laboratories doing serological testing | Nil | University of Otago & Canterbury Health Laboratories; ESR (influenza) | [17, 18] |
| **Respiratory infections** | | | | | | | | |
| ***Myco-bacterium tuberculosis* typing (to support OB detection)** | Incidence of closely related strains of *M. tuberculosis* | Control-focused + Strategy-focused | All specimens of *M. tuberculosis* nation-wide | Event surveillance | Clinical Microbiology Laboratory at LabPlus | Nil | ESR and Auckland LabPlus | [19, 20] |
| **Infections from close physical contact** | | | | | | | | |
| **See NZPSU – Acute Post-Streptococcal Glomerulonephritis** | | | | | | | | |
| **Sexually transmitted infections** | | | | | | | | |
| **HIV/AIDS surveillance** | AIDS cases, HIV positive laboratory diagnoses | Strategy-focused | Total population based on those presenting with AIDS-defining diseases or for HIV testing | Monitoring | AIDS: Medical practitioners. HIV positives: Reference laboratories (ESR Blood borne Virus Lab & Auckland Hospital Virus Lab) | AIDS: Public health service / Medical Officer of Health | AIDS Epidemiology Group (University of Otago) | [3, 21] [22] |
| **Unlinked anonymous HIV prevalence surveys in sexual health clinic attendees** | HIV infection | Strategy-focused | STI clinic attendees, 5+ year intervals | Prevalence surveys | Clinic attendees completing questionnaire | Nil | AIDS Epidemiology Group (University of Otago) | [22] |
| **Clinic-based STI surveillance** | Diagnosed STIs (including  *Chlamydia* infection, gonorrhoea, genital warts, genital herpes, infectious syphilis, non-specific urethritis) | Strategy-focused | Patients who attend participating clinics across NZ | Monitoring | Sexual health clinics, Family planning clinics, Student & youth health clinics | Nil | ESR (Population and Environmental Health Group) | [23] |
| **Laboratory-based STI surveillance** | Positive laboratory test results for STIs (*Chlamydia* infection, gonorrhoea) | Strategy-focused | Patients who attend a healthcare provider who requests a STI test from participating laboratory | Monitoring | Clinical laboratories in most regions of NZ | Nil | ESR (Population and Environmental Health Group) | [23] |
| **Congenital and perinatal infections** | | | | | | | | |
| **See NZPSU – congenital rubella, perinatal HIV exposure, neonatal infections** | | | | | | | | |
| **Blood and tissue borne infections** | | | | | | | | |
| **Haemo-vigilance Programme (surveillance of blood and transfusion-related adverse events)** | HIV, hepatitis B and C in blood donations and transfusion-transmitted infections | Control-focused + Strategy-focused | Blood donors, transfused patients | Event surveillance (reaction reporting), Screening, (blood donors) | Blood banks and hospital clinical staff (DHBs and private hospitals) | DHB blood banks | NZ Blood service (NZBS) | [24] |
| **National Hepatitis Follow-up Programme** | Hepatitis B and C carriage | Control-focused + Strategy-focused | Total population | Screening | Medical practitioners and individuals wanting to be tested | Nil | Hepatitis Foundation of NZ | [25, 26] |
| **Serosurvey and behaviour risk factor survey of Injecting Drug Users (IDUs)** | HIV, hepatitis B and C in IDUs using needle exchange programme (NEP) and knowledge, attitudes and practices | Strategy-focused | IDUs using NEP, 3 year intervals | Prevalence surveys | IDUs interviewed at NEP sites | Nil | NZ Needle Exchange Programme (NZNEP) | [27, 28] |
| **Serological testing of new prison inmates** | HIV, hepatitis B and C | Control-focused | New prison inmates | Screening | Prison medical services | Nil | Department of Corrections | [29] |
| **Creutzfeldt-Jakob disease (CJD) surveillance** | All forms of CJD with focus on variant CJD which may be food borne | Control-focused + Strategy-focused | Total population | Event surveillance | Medical practitioners | Public health service / Medical Officer of Health | CJD registry | [3] |
| **Hospital acquired infections (HAIs) and antibiotic resistance** | | | | | | | | |
| **Hospital-acquired infection (HAI) surveillance** | HAI, including blood stream infections (BSI), surgical site infections (SSI) | Control-focused + Strategy-focused | Variable across DHBs, may include hospital inpatients and post-discharge, continuous and intermittent, specific patient groups | Event surveillance and Monitoring | Hospital staff and laboratories | Infection control staff in some hospitals and DHBs | MoH collates reports on healthcare-associated *Staphylo-coccus aureus* bloodstream infection (part of MoH Hospital Benchmark Indicators reporting) | [30] |
| **Laboratory-based surveillance of HAI (for OB detection)** | Bacteria obtained from hospitalised patients (eg, methicillin-resistant *Staphylo-coccus aureus* [MRSA], Vancomycin-resistant Enterococci [VRE]) | Control-focused + Strategy-focused | Patients with suspected HAI, including hospitalised and out-patients | Event surveillance | Hospital laboratories | Infection control staff in some hospitals and DHBs | ESR (Nosocomial Infections Laboratory) | [31] |
| **Healthcare facilities antibiotic resistance surveillance system** | Outbreaks and elevated rates of important antibiotic resistant organisms (eg, MRSA, ESBL) | Control-focused + Strategy-focused | All hospitals (potentially) | Event surveillance | Hospital infection control staff | Nil | ESR (Antibiotic Reference Laboratory) | [32] |
| **Food borne infections** | | | | | | | | |
| **Laboratory-based surveillance of enteric disease (to support OB detection)** | Enteric bacteria (eg, *Salmonella*, VTEC, *Listeria*), and noroviruses obtained from cases & other sources | Control-focused + Strategy-focused | Patients with suspected enteric disease | Event surveillance | Diagnostic laboratories | Nil | ESR (Enteric Ref Lab) ESR (Norovirus Lab) | [33, 34] |
| **Environmental and water borne infections** | | | | | | | | |
| **Laboratory-based surveillance of legionellosis (to support OB detection)** | Legionellosis and Pontiac fever specimens | Control-focused + Strategy-focused | Patients with suspected legionellosis | Event surveillance | Diagnostic laboratories | Nil | ESR (*Legionella* Ref Lab) | [35] |
| **Zoonotic infections** | | | | | | | | |
| **Laboratory-based surveillance of leptospirosis (to support OB detection)** | Leptospirosis specimens | Control-focused + Strategy-focused | Patients with suspected leptospirosis | Event surveillance | Diagnostic laboratories | Nil | ESR (Leptospirosis Ref Lab) / Massey University (Institute of Veterinary, Animal and Biomedical Sciences) | [36] |
| **Vector borne infections** | | | | | | | | |
| **Laboratory-based surveillance of arboviral diseases (to support OB detection)** | Serological specimens from suspect cases (eg, dengue, Ross River virus infection) | Control-focused + Strategy-focused | Patients with suspected arboviral disease | Event surveillance | ESR (for NZ except for Auckland and Northland), LabPlus (Auckland and Northland) | Nil | ESR (Arbovirus Lab) | [37] |
| **New, exotic and imported infections** | | | | | | | | |
| **Laboratory-based surveillance of viruses (to support OB detection)** | Range of respiratory, enteric and contact spread viruses (eg, adenoviruses, enteroviruses) | Control-focused + Strategy-focused | Patients with suspected viral disease | Event surveillance | ESR Virus Identification Reference Lab plus other NZ laboratories (5) doing virological testing | Nil | ESR (Virus Identification Reference Lab) | [38] |
| **Arriving passenger screening** | Arriving passenger with respiratory or other infectious disease symptoms or seated near to a person with symptoms | Control-focused + Strategy-focused | Arriving passengers (particularly during pandemic situations) | Screening | Arriving passengers | Public health service / Medical Officer of Health | MoH (on occasions eg, during an influenza pandemic) | [39] |
| **Quota refugee health screening** | Cases of infectious disease in refugees are reported via the relevant system eg, notifiable disease surveillance | Control-focused + Strategy-focused | All quota refugees | Screening | Auckland Regional Public Health Service (ARPHS) | ARPHS | As for the relevant infectious disease | [40, 41] |
| **Immigrant screening** | Completion of medical and chest x-ray certificates | Control-focused | People who wish to visit, study, or work in NZ and all people who wish to reside here permanently are screened | Screening | Medical examiners in NZ and offshore | Nil | None | [42] |
| **Geosentinel (Global Surveillance Network of the International Society of Travel Medicine and Centres for Disease Control)** | Returning international travellers and migrants with specific infections | Strategy-focused | Returning travellers attending WorldWise Travellers Health and Vaccination Centre, Auckland | Monitoring | General practitioners at WorldWise Travellers Health and Vaccination Centre, Auckland | Nil | No national collation in NZ – collated by Geosentinel network headquarters in US | [43] |
| Surveillance of Hazards | | | | | | | | |
| **Vaccine-preventable infections** | | | | | | | | |
| **See also interventions for vaccine preventable infections (National Immunisation Register, school-based registers, coverage surveys)** | | | | | | | | |
| **Laboratory-based surveillance of invasive bacterial diseases (to support vaccine strategy)** | Vaccine-preventable invasive bacteria (Pneumococci, Meningococci, *Haemophilus*) | Strategy-focused | Patients with suspected invasive bacterial disease | Monitoring | Diagnostic laboratories | Nil | ESR (Invasive Pathogen Lab) | [44] |
| **Laboratory-based surveillance of influenza (to support vaccine strategy and outbreak detection during pandemics)** | Influenza virus | Strategy-focused | Total population | Monitoring, Event surveillance (start of pandemics) | Diagnostic laboratories, GP Sentinel Surveillance System (see above), Hospital laboratories | Nil | ESR (Virus Identification Ref Lab) | [11, 12] |
| **Surveillance of influenza knowledge, attitudes and practices** | Knowledge, attitudes and practices relevant to reducing person risk of acquiring influenza or transmitting it to others | Strategy-focused | A sample of the population | Prevalence surveys | Directly from interview subjects or by observation | Nil | Research providers | [45, 46] |
| **Respiratory infections** | | | | | | | | |
| **Tobacco use surveillance (including active smoking and exposure to second-hand smoke (SHS))** | Active smoking and exposure to SHS | Strategy-focused | Total population | Prevalence surveys (Census, National Health Survey), Monitoring (imports and sales) | Adult smokers and non-smokers and various sources eg, Customs data | Nil | MoH, Statistics NZ | [47] |
| **Infections from close physical contact** | | | | | | | | |
| **No specific systems** | | | | | | | | |
| **Sexually transmitted infections** | | | | | | | | |
| **HIV behavioural surveillance among men who have sex with men (MSM)** | Sexual partnering, sexual behaviours, condom use, HIV testing, HIV status, STI diagnosis, and attitudes towards HIV and safe sex | Strategy-focused | The Gay Auckland Periodic Sex Survey (GAPSS) based on Auckland sample of MSM; Gay men’s Online Sex Survey (GOSS) based on nationwide Internet sample of MSM | Prevalence surveys | NZ AIDS Foundation and AIDS Epidemiology Group | Nil | NZ AIDS Foundation and the AIDS Epidemiology Group | [48] |
| **HIV Futures New Zealand** | Sexual behaviours, attitudes towards HIV | Strategy-focused | Sample of HIV positive people | Prevalence surveys | NZ AIDS Foundation and AIDS Epidemiology Group | Nil | NZ AIDS Foundation and AIDS Epidemiology Group | [49] |
| **Congenital and perinatal infections** | | | | | | | | |
| **Antenatal infectious disease screening (HIV, rubella, Hepatitis B virus (HBV), tuberculosis risk)** | Positive test results in pregnant women (HBV, HIV) and infants at high risk of tuberculosis | Control-focused | Pregnant women seen by a health professional (medical practitioners, midwives) | Screening | Hospital specialist medical practitioners | DHBs | Antenatal HIV screening data collated by National Screening Unit and AIDS Epidemiology Group | [50, 51] |
| **Blood and tissue borne infections** | | | | | | | | |
| **See also haemovigilance, Hepatitis B & C** | | | | | | | | |
| **Registration of skin piercing practitioners** | Skin piercing practices (beauticians, tattooists, skin piercers) | Control-focused | All skin piercing practitioners | Service tracking | Skin piercing practitioners when inspected and interviewed | Local authority | None | [52] |
| **Needle-stick injuries surveillance** | Needle-stick injuries | Control-focused | All DHBs | Event surveillance | Healthcare workers following needle-stick injury | Infection control and occupational health staff in some hospitals and DHBs | None | NI |
| **Hospital-acquired infections and antibiotic resistance** | | | | | | | | |
| **Identification of hospital patients colonised with high-risk organisms** | Patients colonised with medically important bacteria (eg, MRSA, ESBL, VTEC, *Clostridium difficile*) | Control-focused | Population admitted to hospital (some DHBs) | Screening | Hospital staff and laboratories | Infection control staff in some hospitals and DHBs | No national collation, Individual patients can be identified through an alert on the National Health Index number | [30] |
| **Surveillance of hospital operative equipment** | Contaminated equipment eg, endoscopes, 'single-use' items being reused | Control-focused | A sample of hospital equipment (some DHBs) | Screening | Hospital staff and laboratories | Infection control staff in some hospitals and DHBs | None | [30] |
| **Hand Hygiene New Zealand (HHNZ)** | Hand washing behaviour | Strategy-focused | Some DHBs | Prevalence surveys | Hospital infection control staff | Infection control staff in some hospitals and DHBs | National Quality Improvement Programme (NQIP) | [53] |
| **Antimicrobial resistance surveillance** (several systems to collect national antimicrobial resistance data) | Antibiotic resistance among medically important bacteria in NZ | Strategy-focused | Bacteria of interest identified by labs are referred to the Antibiotic Reference Laboratory, which also collects selected data from other laboratories | Monitoring, and Prevalence surveys | Hospital and community diagnostic laboratories, Reference laboratories (ESR) | Nil | ESR (Antibiotic Reference Laboratory) | [32] |
| **Surveillance of antibiotic prescribing to humans** | Antibiotic prescribing | Strategy-focused | Antibiotic prescriptions through Pharma-ceutical Benefits Scheme | Monitoring | Prescription data collated by MoH | Nil | Pharmac, Best Practice Advocacy Centre (BPAC) | [54] |
| **Food borne infections** | | | | | | | | |
| **See also interventions for food borne infections (Processed and retail food surveillance and auditing of food control plans)** | | | | | | | | |
| **Surveillance of antibiotic resistance in food animals** | Antibiotic resistant bacteria in food-producing animals | Strategy-focused | A sample of food-producing animals | Prevalence surveys | New Zealand Food Safety Authority (NZFSA) | Nil | NZFSA | [55] |
| **Surveillance of veterinary medicine (antibiotic) sales** | Sale of prescription veterinary antibiotics | Strategy-focused | All prescription antibiotics | Monitoring | NZFSA | Nil | NZFSA | [56] |
| **Imported Foods Monitoring Programmes (IFMP)** (monitoring hazards in imported food)**; Imported Foods Clearance Programmes** (monitoring compliance at the border) | IFMP: determining trends and monitoring the effectiveness of controls; IFCP: detection of selected hazards in high risk imported foods | Control-focused + Strategy-focused | A sample of imported food | Screening (IMCP), Monitoring (IFMP) | NZFSA | Nil | NZFSA and Ministry of Agriculture and Forestry Biosecurity NZ (MAFBNZ) | [57] |
| **National Microbial Database (NMD) (food production/ processing surveillance)** | Pathogens in carcasses of specified food animal species presenting for food processing | Control-focused + Strategy-focused | A sample of carcasses at food processing/ production plants | Screening | Individual food production companies | Nil | NZFSA | [58] |
| **Surveillance of marine biotoxins (Non-Commercial Marine Biotoxin Monitoring Programme; Commercial Shellfish Monitoring)** | Levels of marine biotoxins of public health concern in shellfish | Control-focused + Strategy-focused | A sample of shellfish and marine settings | Screening | Public health services and other agencies | Public health services and other agencies | NZFSA | [59] |
| **Food handling knowledge, attitudes and practices surveys** | Reported food safety knowledge, attitudes and practices | Strategy-focused | Population sample, variable content and intervals | Prevalence surveys | Interview subjects | Nil | NZFSA | [60] |
| **Environmental and water borne infections** | | | | | | | | |
| **See also interventions for environmental and water borne infections (Drinking water surveillance)** | | | | | | | | |
| **Natural recreational water surveillance** | Indicator organisms in recreational waters | Control-focused + Strategy-focused | Selected swimming beaches (coastal and inland) usually sampled weekly during the swimming season | Screening | Local authorities | Local authorities (may share findings with public health services) | Ministry for the Environment and MoH | [61] |
| **Cooling tower surveillance** | Management of cooling towers and *Legionella* contamination levels | Control-focused | All cooling towers (potentially) | Screening | Cooling tower owner plus ESR and commercial laboratories (for *Legionella* testing) | Local public health services | None | [62] |
| **Public swimming pool surveillance** | Management of water quality and monitoring of indicator organisms guided by the Pool Water Quality Standards | Control-focused | All public swimming pools (potentially) | Screening | Pool owner | Local public health services (in response to outbreaks or routine auditing) | None | NI |
| **Zoonotic infections** | | | | | | | | |
| **OIE Notifiable Organisms surveillance** (OIE is the world organisation for animal health) | OIE notifiable organisms in animals including wildlife | Control-focused + Strategy-focused | All animals and appropriate settings | Event surveillance | Department of Conservation, Massey University, Auckland Zoo, Veterinary laboratories, veterinarians, general public | Nil | MAFBNZ | [63] [64] |
| **Veterinary laboratory-based surveillance** | All animal diseases | Control-focused + Strategy-focused | Livestock or symptomatic animals | Event surveillance and Monitoring | MAFBNZ approved veterinary diagnostic laboratories | Nil | MAFBNZ | [65] |
| **Avian influenza surveillance** | Birds infected and dying with highly pathogenic notifiable avian influenza (HPNAI) or notifiable avian influenza (NAI) | Control-focused + Strategy-focused | All birds (including commercial poultry) and samples of migratory shorebirds and resident waterfowl | Event surveillance | MAFBNZ | Nil | MAFBNZ | [66] |
| **Transmissible spongiform enceph-alopathy (TSE) disease surveillance** | Livestock infected with TSE (including bovine spongiform encephal-opathy – BSE) | Control-focused + Strategy-focused | Susceptible livestock showing symptoms consistent with TSE disease | Event surveillance | MAFBNZ and NZFSA | Nil | Nationally: MAFBNZ, NZFSA | [67] |
| **Bovine tuberculosis surveillance** | Incidence of infected animals and operation of vector control programmes | Strategy-focused | All livestock and a sample of vectors | Event surveillance and Prevalence surveys | Veterinarians and Animal Health Board staff | Nil | Animal Health Board | [68] [69] |
| **Vector borne infections** | | | | | | | | |
| **Arbovirus surveillance** | Serological detection of specific arboviruses in cattle and other animals | Control-focused + Strategy-focused | A sample of cattle and other animals | Prevalence surveys and Monitoring | Active surveillance by MAFBNZ staff | Nil | MAFBNZ | [70, 71] |
| **Mosquito surveillance** | Identification of imported mosquitoes at ports; identification of exotic mosquitoes in salt marshes and suitable saltmarsh mosquito habitats. | Control-focused + Strategy-focused | A sample of imported mosquitoes and saltmarsh mosquitoes and habitats | Screening | Public health services , the saltmarsh mosquito surveillance system and NZ BioSecure Entomology Laboratory (NZBEL) | Nil | MAFBNZ, MoH | [72, 73] |
| **New, exotic and imported infections** | | | | | | | | |
| **Border surveillance** | Detection of pests, diseases and unwanted organisms entering NZ | Control-focused + Strategy-focused | Samples of livestock, germplasm, animal products and horticultural produce imported into NZ | Event surveillance, Screening | Variety of different agencies (primarily MAFBNZ and NZFSA Verification Agency) | Nil | MAFBNZ and NZFSA | [74] |
| Surveillance of Determinants | | | | | | | | |
| **Population size and characteristics** | | | | | | | | |
| **Census** | Size and demographic characteristics of NZ population and sub-populations | Strategy-focused | Total population | Prevalence surveys | Census enumerator | Nil | Statistics NZ | [75] |
| **NZ Health Tracker** | Size & characteristics of NZ population with chronic disease | Strategy-focused | Total population | Monitoring | Derived from linking multiple health datasets (including NMDS, Pharmhouse, Laboratory, Primary Health Organisation enrolments) | Nil | MoH | NI |
| **The Maternity and New-born Collection** | Maternal and new-born population | Strategy-focused | Total population up to 9 months before and 3 months after birth | Monitoring | HealthPac maternity claims system | Nil | MoH | [76] |
| **New birth registration** | Incidence of births and still-births | Strategy-focused | Total population | Monitoring | Hospitals, doctors, midwives | Nil | Births, Deaths and Marriages and Statistics NZ | [77] |
| **Healthcare worker registrations** | Number, occupational group and work hours of healthcare workers | Strategy-focused | Healthcare workers | Monitoring | Healthcare workers who will all require a Health Practitioner Index (HPI) | Nil | MoH, registration agencies (eg NZ Medical Council, Nursing Council) | [78, 79] |
| **Arriving passenger survey** | Volume of travelling population and travel history | Strategy-focused | Sample of arriving passengers | Prevalence surveys | NZ Customs Service | Nil | Statistics NZ | [80] |
| **Other determinants** | | | | | | | | |
| **Surveillance of socio-economic conditions** | A set of socioeconomic position statistical indicators | Strategy-focused | Represent-ative population (national censuses and surveys) | Prevalence surveys | Various governmental and other agencies which run nation-wide surveys/ censuses | Nil | Ministry of Social Development, Statistics NZ, MoH | [81-83] |
| **Environ-mental health indicator surveillance** | Environmental hazards and conditions that affect health | Strategy-focused | Multiple environmental settings which may have health effects | Monitoring and Prevalence surveys | ESR Laboratories, National Institute of Water & Atmospheric Research, MoH, National Radiation Laboratory, Land Transport Safety Authority, and local authorities | Nil | MoH | [84] |
| **Surveillance of health service conditions** | Statistical indicators of health-sector performance | Strategy-focused | Represent-ation of total population (national censuses and surveys) | Monitoring and Prevalence surveys | Various governmental and other agencies which run nation-wide surveys/ censuses | Nil | MoH | [85] |
| Surveillance of interventions | | | | | | | | |
| **Interventions for vaccine-preventable infections** | | | | | | | | |
| **National Immunisation Register (NIR) (immunis-ation coverage)** | Vaccination status of children on the NIR, coverage of publicly funded vaccines | Control-focused + Strategy-focused | All children born after 1/12/2005 are registered on the NIR. Some children born before this date are registered if they have received a NIR notifiable vaccine such as MeNZB | Service tracking | General practitioners (electronically via patient management systems) and other vaccination providers | Public health services can access and use | MoH - NIR | [86, 87] |
| **Immunisation coverage surveys** | Coverage of publicly-funded vaccines | Strategy-focused | Represent-ative sample of NZ children | Prevalence surveys | Parents and general practitioners | Nil | MoH | [86, 88] |
| **School-based immunisation registers (immunis-ation coverage)** | Coverage of publicly-funded vaccines given to children born from 1/1/1995 who attend early childhood services and primary schools | Control-focused + Strategy-focused | Children attending primary schools and early childhood centres (ECC) | Service tracking | All ECCs and primary schools | Retained by schools and ECCs | None | [89] |
| **Influenza vaccine surveillance** | Prevalence of individuals vaccinated against influenza | Strategy-focused | Those over 65 years old and with specified chronic diseases | Monitoring | Benefits claim data and private pharma-ceutical sales | Nil | MoH | [11] |
| **Adverse event surveillance** | Adverse reactions to vaccines (and also medicines, herbal products and dietary supplements) | Strategy-focused | Total population following vaccination | Monitoring | Health professionals | Nil | Centre for Adverse Reactions Monitoring, Intensive Medicines Monitoring Programme (IMMP), MedSafe, NIR | [90, 91] |
| **Cold-chain surveillance** | Efficacy of vaccine storage by providers of immunisation; incidence of breaks in the cold-chain. | Control-focused + Strategy-focused | A sample of vaccines | Service tracking | Cold-chain accreditation reviews and national cold-chain audits | Regional immunisation advisors / Public health service | ESR and the Immunisation Advisory Centre (IMAC) | [92, 93] |
| **Interventions for other infections diseases** | | | | | | | | |
| **Rheumatic fever registers** | People notified with acute rheumatic fever to support long-term prophylaxis | Control-focused + Strategy-focused | All notified cases of rheumatic fever | Service tracking | Medical practitioners (usually paediatricians) | Public health service / Medical Officer of Health | None | [94] |
| **Surveillance of contact prophylaxis and treatment** | Contact investigation and treatment (influenza, being extended to other diseases) | Control-focused | Selected diseases (influenza nationally, other diseases in some regions). All contacts considered at risk | Service tracking | Public health services staff managing case investigation | Public health service / Medical Officer of Health | None | NI |
| **Processed and retail food surveillance (being replaced by auditing of food control plans – see below)** | Compliance with the Food Hygiene Regulations 1974 | Control-focused + Strategy-focused | All registered premises selling food | Service tracking | NZFSA and Environmental health officers (EHOs) | Local authorities | NZFSA | [95] |
| **Surveillance of risk management programmes and food control plans** | Suitable food control plans for food premises and production | Control-focused + Strategy-focused | All food control plans | Service tracking | Individual food premises and production companies | NZFSA and Local authorities | NZFSA | [95] |
| **Drinking water surveillance** | Compliance with Drinking Water Standards, including indicator organisms | Control-focused + Strategy-focused | Samples of drinking water taken by water suppliers, Health Protection Officers | Screening | Water supply owners (eg, local authorities), Health Protection Officers | Public health services | Water Information System NZ (WINZ); ESR database (annual survey of compliance with Drinking Water Standards) | [96] |

**Key to table**

**System name (or subject area)**

- Name of surveillance system or subject area if the system doesn’t have a specific name

#### Event under surveillance

- Disease – including injury, changes in health status, health outcomes
- Hazards – risk and protective factors (including behaviours, population vulnerability, agent characteristics, and exposures) that may affect health through specific, direct causal mechanisms
- Determinants – causal factors that may affect health through multiple, often indirect pathways
- Interventions – actions taken to control or prevent the occurrence of disease or minimise its negative health effects

#### Main purpose – Based on the stated or implied purpose of the current system

- Control-focused – surveillance that aims to identify each occurrence of a particular disease, hazard or other health-related event that requires a specific response and support delivery of an effective intervention
- Strategy-focused – surveillance that provides information to support prevention strategies to reduce population health risk. Prevention may precede disease (primary prevention) or be focused on reducing the effects of disease (secondary and tertiary prevention)
- Control-focused and strategy-focused – surveillance often meets both purposes as control-focused surveillance usually provides information that can also be used for strategy-focused surveillance purposes

#### Coverage – Population or Setting

- A description of the population or setting covered by the surveillance system and the completeness of that coverage

#### System type – Based on the type of current system

- Event surveillance - prospective surveillance to identify in a timely manner each occurrence of a particular health-related event, including disease and injury cases, outbreaks, health hazards and interventions
- Screening - surveillance to identify a particular inapparent disease or pre-disposing risk factor in all members of a specified population, or a particular health hazard in specified settings or environments
- Service tracking - surveillance to identify delivery and non-delivery of a particular intervention or programme of agreed quality to specified individuals, populations and settings
- Prevalence surveys – surveillance based on repeated surveys to measure the prevalence over time of a particular disease or injury, health state, health hazard, health determinant or intervention use in a specified population or setting
- Monitoring - surveillance based on collection and periodic analysis and interpretation of information to characterise the occurrence and distribution of a particular health-related event, including disease and injury cases, health states, health hazards, determinants and interventions.

#### Reporting source

- The point at which the health-related event or state is first detected and recorded by the surveillance system

#### Local/ Regional collation

- The point at which information generated by the surveillance system is collected, analysed, and used at an institutional or regional level, often to guide prevention and control measures

#### National collation

- The point at which information generated by the surveillance system is collected, analysed, and used at a national level, often to guide prevention policies and programmes

#### Reference(s)

- Sources that provide a description of the surveillance system, including published and on-line information sources. In some cases no appropriate documentation was identified as indicated by “NI” (for “not identified”) and information from key informants and/or knowledge of the authors was used instead

**References for additional file 2**
